# Evaluation of Whole-Tumor Texture Analysis Based on MRI Diffusion Kurtosis and Biparametric VI-RADS Model for Staging and Grading Bladder Cancer

**DOI:** 10.3390/bioengineering10070745

**Published:** 2023-06-21

**Authors:** Xiaoyan Meng, Shichao Li, Kangwen He, Henglong Hu, Cui Feng, Zhen Li, Yanchun Wang

**Affiliations:** 1 Department of Radiology, Tongji Hospital, Tongji Medical College, Huazhong University of Science and Technology, Wuhan 430030, China; xymeng@hust.edu.cn (X.M.); zhenli@hust.edu.cn (Z.L.); 2 Department of Urology, Tongji Hospital, Tongji Medical College, Huazhong University of Science and Technology, Wuhan 430030, China

**Keywords:** bladder cancer, diffusion kurtosis imaging, texture analysis, staging, grading

## Abstract

Background: to evaluate the feasibility of texture analysis (TA) based on diffusion kurtosis imaging (DKI) in staging and grading bladder cancer (BC) and to compare it with apparent diffusion coefficient (ADC) and biparametric vesical imaging reporting and data system (VI-RADS). Materials and Methods: In this retrospective study, 101 patients with pathologically confirmed BC underwent MRI with multiple-b values ranging from 0 to 2000 s/mm^2^. ADC- and DKI-derived parameters, including mean kurtosis (MK) and mean diffusivity (MD), were obtained. First-order texture histogram parameters of MK and MD, including the mean; 5th, 25th, 50th, 75th, and 90th percentiles; inhomogeneity; skewness: kurtosis; and entropy; were extracted. The VI-RADS score was evaluated based on the T2WI and DWI. The Mann–Whitney U-test was used to compare the texture parameters and ADC values between non-muscle-invasive bladder cancer (NMIBC) and muscle-invasive bladder cancer (MIBC), as well as between low and high grades. Receiver operating characteristic analysis was used to evaluate the diagnostic performance of each significant parameter and their combinations. Results: The NMIBC and low-grade group had higher MDmean, MD5th, MD25th, MD50th, MD75th, MD90th, and ADC values than those of the MIBC and the high-grade group. The NMIBC and low-grade group yielded lower MKmean, MK25th, MK50th, MK75th, and MK90th than the MIBC and high-grade group. Among all histogram parameters, MD75th and MD90th yielded the highest AUC in differentiating MIBC from NMIBC (both AUCs were 0.87), while the AUC for ADC was 0.86. The MK75th and MK90th had the highest AUC (both 0.79) in differentiating low- from high-grade BC, while ADC had an AUC of 0.68. The AUC (0.92) of the combination of DKI histogram parameters (MD75th, MD90th, and MK90th) with biparametric VI-RADS in staging BC was higher than that of the biparametric VI-RADS (0.89). Conclusions: Texture-analysis-derived DKI is useful in evaluating both the staging and grading of bladder cancer; in addition, the histogram parameters of the DKI (MD75th, MD90th, and MK90th) can provide additional value to VI-RADS.

## 1. Introduction

Bladder cancer (BC) is one of the most common tumors in the urinary tract [1]. The most common pathological type of bladder cancer is urothelial carcinoma, and treatment depends on the depth of muscle invasion and the tumor grade [2,3]. Transurethral resection of bladder tumor (TURBT) is the primary treatment for patients with low-grade non-muscle-invasive bladder cancer (NMIBC) [2], while radical cystectomy, adjuvant chemotherapy, or both are the primary treatment for patients with high-grade NMIBC or MIBC [3]. Therefore, it is particularly important for patients and urologists to distinguish the stage and grade of bladder cancer. TURBT is a routine method for clinically determining the muscle-invasiveness and histological grade of bladder cancer [2,3]. However, for MIBCs, approximately 25% of cases are underestimated and about 15% of tumor histological grades may be inaccurate because of specimen errors if insufficient detrusor ranges are collected in excised specimens [4,5]. Because of the above limitations, as well as the high cost and invasiveness of TURBT, there is an urgent need for a non-invasive and accurate imaging method to evaluate the stage and grade of bladder cancer.

With the advantage of the high resolution of soft tissue, magnetic resonance imaging (MRI) has been widely used in the diagnosis of bladder cancer as a non-invasive imaging method [6]. In 2018, the vesical imaging reporting and data system (VI-RADS) was introduced as reporting standards for bladder MRI to reduce inconsistencies among readers and improve diagnostic accuracy [7]. Currently, many studies have confirmed that VI-RADS has high diagnostic accuracy in the diagnosis of muscle invasiveness of bladder cancer, but it is still a semiquantitative subjective scoring system.

As indicated by the recently developed VI-RADS [7], the role of DWI in staging bladder cancer has become more important. The apparent diffusion coefficient (ADC), obtained from diffusion-weighted imaging (DWI) based on a mono-exponential diffusion model, can provide useful diagnostic information for grading and staging bladder tumors [8,9,10]. In addition, ADC has been introduced as a quantitative parameter for the characterization of bladder cancer, but its clinical application has been hindered by the relatively large overlap of ADC values [11]. This overlap may be attributed to the inability of a mono-exponential diffusion model reflecting the Gaussian motion of water molecules in tissues to fully explore the underlying microstructural complexity of bladder cancer. Diffusion kurtosis imaging (DKI) can reflect the non-Gaussian diffusion, the complexity and heterogeneity of the tissue and cell microstructure, and provide more information than conventional DWI [12]. DKI has been successfully used to grade bladder cancer and assess its muscle invasiveness [13,14]. The DKI (MK) parameter had a better diagnostic performance than conventional DWI in predicting muscle invasiveness [14]. Moreover, kurtosis metrics performed well at differentiating MIBC from NMIBC [13].

Texture analysis (TA) has become a widely used method in recent years for post-processing image data, which can be applied retrospectively to medical images to assess tumor complexity, heterogeneity at a subvoxel level, and obtain more lesion information. TA has certain value in determining tumor subtypes and evaluating the biological characteristics of tumors, also it has been used for predicting the histological grade and muscle invasiveness of BC with certain diagnostic value based on CT and MR images [15,16,17]. To our knowledge, few studies have demonstrated the feasibility of TA based on DKI in assessing the stage and grade of BC preoperatively.

Therefore, the purpose of this study was to investigate the potential of histogram parameters derived from DKI in predicting the stage and histologic grade of BC, and compare their diagnostic efficacy with ADC and biparametric VI-RADS.

## 2. Materials and Methods

### 2.1. Patient Characteristics

**Figure 1:**
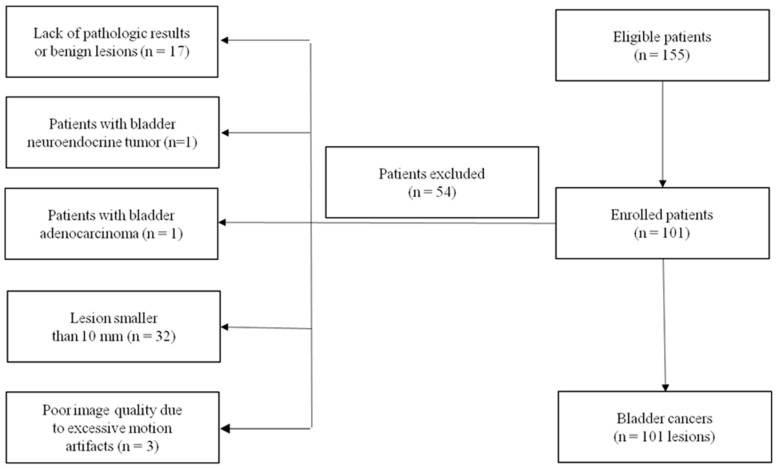
Flowchart of the study population.

This retrospective study was approved by the ethics committee of our hospital, and the requirement for informed consent was waived. Between August 2014 and July 2021, 155 patients with suspected bladder cancer who underwent MRI examination were enrolled. The inclusion criteria were as follows: (1) histopathological confirmed bladder cancer; (2) lesions equal to or greater than 10 mm; (3) no history of radiotherapy, chemotherapy, or TURBT within two weeks before MRI; (4) adequate image acquisition and quality without sensitive artifacts or patient movement motion. A total of 54 patients were excluded because of the following reasons: (1) lack of pathological results (n = 5); (2) pathological diagnosis of inflammatory lesions (n = 12); (3) diagnosis as adenocarcinoma of bladder (n = 1) or neuroendocrine tumor of bladder (n = 1); (4) flattened or insufficient tumor area (diameter < 10 mm), and the VOIs could not be mapped (n = 32); (5) poor image quality due to excessive motion artifacts (n = 3). Finally, a total of 101 patients (87 males and 14 females; median age = 63.5 ± 9.8 years; age range = 36–84) were included in this study. The flowchart of the study population is shown in Figure 1.

### 2.2. Image Acquisition

All participants underwent MR examinations in a 3.0 T scanner (Discovery MR750; GE Healthcare, Milwaukee, MI, USA) with a 32-channel torso phased-array coil in the supine position. The imaging protocols, including axial fast spin-echo (FSE) T1-weighted imaging (T1WI), axial fast recovery FSE T2-weighted imaging (T2WI), sagittal FSE T2-weighted imaging (T2WI), and multiple *b* value DWI, were acquired. The acquisition parameters of each non-diffusion imaging sequence were as follows: (1) axial T1WI—repetition time/echo time = 528/6.8 ms, field of view (FOV) = 340 × 340 mm^2^, matrix size = 320 × 256, and echo train length = 4; (2) axial fast recovery T2WI—repetition time/echo time = 3780/75 ms, FOV = 340 × 340 mm^2^, matrix size = 320 × 256, and echo train length = 16; (3) sagittal T2WI—repetition time/echo time = 5500/75 ms, FOV = 240 × 240 mm^2^, matrix size = 320 × 320, and echo train length = 24. In all sequences above, a section thickness of 4 mm with an intersection gap of 1 mm was used together with 2 averages. A series of axial DWI were acquired using a SSEPI sequence with 13 *b* values (b_NEX_): 0_1_, 50_1_, 80_1_, 100_1_, 150_1_, 200_1_, 300_2_, 500_2_, 800_2_, 1000_4_, 1300_4_, 1700_6_, and 2000_6_ s/mm^2^. At each non-zero b value, a Stejskal–Tanner diffusion gradient was successively applied along the three orthogonal directions to obtain trace-weighted images to minimize the influence of diffusion anisotropy. The additional acquisition parameters for the DWI sequence were as follows: repetition time/echo time = 2500/84 ms, field of view = 400 × 400 mm^2^, matrix size = 128 × 160, section thickness = 4 mm, intersection gap = 1 mm, and a scan time of approximately 4 min.

### 2.3. Image Processing and Analysis

For the patients with multifocal lesions, only the tumor with the higher tumor burden (larger diameter or higher stage) was analyzed in this study. The diffusion-weighted images of each patient were analyzed with kurtosis model and mono-exponential model using a freely available FireVoxel software package (NYU Center for Advanced Imaging Innovation and Research, New York, NY, USA) on a personal computer. Volumetric histogram analysis of DKI was performed by the two radiologists (X. Y. M. and Y. C. W., with 8 and 10 years of experience in clinical MRI diagnosis, respectively). For each patient, the volume of interest (VOI) was drawn covering the entire lesion with appropriate size on all continuous slices of the tumors on DWI at *b* value of 1000 s/mm^2^. As the low-signal pedicle may be related to the extent of tumor invasion, the stalks with low-signal intensity on DWI were also included in the VOIs [18,19,20]. Finally, The ADC maps within VOI were calculated automatically with FireVoxel software using a DKI model:

S(*b*) = S_0_ exp (−*b*MD*_b_* + *b*^2^MD*_b_*^2^MK*_b_*/6) (1)

where S(*b*) is the DWI signal intensity at a specified *b* value, S_0_ is the baseline signal at *b* = 0, and the *b* is known as the *b* value. MK*_b_* is a dimensionless metric representing the deviation of water motion from Gaussian distribution, and MD*_b_* is the kurtosis corrected diffusion coefficient. The texture parameters derived from MK and MD were generated automatically using the FireVoxel software, including the mean; 5th, 25th, 50th, 75th, and 90th percentiles; inhomogeneity; skewness; kurtosis; and entropy.

ADC maps were also automatically calculated via the mono-exponential model according to the following equation:

S(*b*) = S_0_ exp∙(−*b*ADC) (2)

where S(*b*) is the signal in the presence of diffusion at a specified *b* value, and S_0_ is the signal in the absence of diffusion. The *b* is known as the *b* value, which determines the degree of diffusion motion weighting in the signal.

### 2.4. VI-RADS Scoring

Because dynamic contrast-enhanced (DCE) MRI was not available for all patients (18 patients did not have DCE sequences), biparametric VI-RADS score was calculated independently by the radiologists (X. Y. M. and Y. C. W.) using T2WI and DWI sequences without being informed of the histopathological findings. Each selected tumor was scored based on the five-point VI-RADS scoring system (see Supplementary Materials Figure S1) to score these two sequences (T2WI and DWI), as described in previous studies [7], and a VI-RADS score was assigned for each patient.

### 2.5. Statistical Analysis

Statistical analysis was performed using SPSS (version 24.0, IBM, Armonk, NY, USA), and MedCalc (version 12.7, MedCalc Software, Ostend, Belgium). Clinical and statistical data are presented as the mean ± standard deviation (SD). All tests were two-sided and *p*-value < 0.05 was considered statistically significant. The intraclass correlation coefficient (ICC) was used to compare the consistency between two readers, and the agreement was interpreted according to the ICC (excellent consistency, ICC > 0.80; good consistency, ICC 0.61–0.80; moderate consistency, ICC 0.41–0.60; general consistency, ICC 0.21–0.40; poor consistency, ICC ≤ 0.20). Mann–Whitney U tests were used for the comparison of each volumetric MD, MK histogram parameters, and ADC values between the NMIBC and MIBC groups and between the low- and high-grade groups. Receiver operating characteristic (ROC) analysis was performed to evaluate the diagnostic performance of each parameter in predicting aggressiveness. Meanwhile, the corresponding area under the ROC curve (AUC) with 95% confidence intervals (95% CI) was calculated, and the optimum cut-off value was determined according to the Youden index.

## 3. Results

### 3.1. Clinical Characteristics

**Figure 2:**
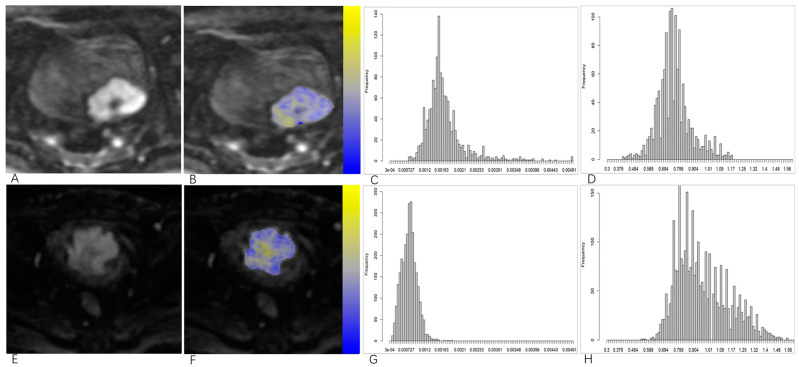
Upper row: a 63-year-old man with bladder cancer (papillary urothelial carcinoma, category T1, low grade): (**A**) axial DWI images; (**B**) regions of interests (ROIs) of the tumor area; (**C**) volumetric MD histogram; (**D**) volumetric MK histogram. Lower row: a 74-year-old man with bladder cancer (papillary urothelial carcinoma, category T2, high grade): (**E**) axial DWI images; (**F**) regions of interests (ROIs) of the tumor area; (**G**) volumetric MD histogram; (**H**) volumetric MK histogram.

The clinical characteristics of the patients are summarized in Table 1. Among the 101 patients, 32 underwent radical or partial bladder resection, and 69 underwent TURBT. According to the eighth version of TNM system [21], 67 patients with NMIBC (37 cases of Ta stage and 30 cases of T1 stage) and 34 patients with MIBC (28 cases of T2 stage, 2 cases of T3 stage, and 4 cases of T4 stage) were determined by pathological T-staging. According to the 2016 World Health Organization classification system [22], 31 patients had tumors classified as high grade. The representative images of NMIBC and MIBC are presented in Figure 2.

### 3.2. Interobserver Agreement

The consistency of measurements between the two observers was evaluated by ICC, and the results presented that all texture features of MD (ICC 0.900–0.997), MK (ICC 0.915–0.998), and ADC (ICC 0.910–0.996) had good consistency (see Supplementary Materials Table S1).

### 3.3. Comparisons of Volumetric DKI Histogram Parameters and ADC Values between the NMIBC and MIBC Groups and Low- and High-Grade Groups

The comparison of MD and MK histogram parameters between the MIBC and NMIBC groups, and between low- and high-grade groups were shown in Table 2 and Table 3. The MDmean, MD5th, MD25th, MD50th, MD75th, and MD90th in the NMIBC group were higher than those in the MIBC group (all *p* < 0.001). However, the MDskewness and MDkurtosis were lower in the NMIBC group than in the MIBC group (both *p* < 0.001). There was no significant difference between NMIBC and MIBC in MDinhomogeneity and MDentropy (*p*-value was 0.12 and 0.86, respectively). MKmean, MK25th, MK50th, MK75th, and MK90th in the NMIBC group were lower than those in MIBC group (all *p* < 0.05). However, there was no significant difference between NMIBC and MIBC in terms of MK5th, MKskewness, MKkurtosis, MKinhomogeneity, and MKentropy (*p*-values of 0.99, 0.13, 0.33, 0.52, and 0.25, respectively). MDmean, MD5th, MD25th, MD50th, MD75th, and MD90th in high-grade group were lower than those in low-grade group (all *p* < 0.05). The MDinhomogeneity and MDskewness of the high-grade group were higher than those of the low-grade group (all *p* < 0.05). There was no significant difference in the MDkurtosis and Mdentropy between the low- and high-grade groups (*p*-values were 0.05 and 0.10, respectively). In the high-grade group, the MKmean, MK25th, MK50th, MK75th, MK90th, and MKentropy were higher than in the low-grade group (all *p* < 0.05). However, there were no significant differences in the MK5th, MKinhomogeneity, MKskewness, and MKkurtosis between the low- and high-grade groups (*p*-values were 0.14, 0.64, 0.06, and 0.17, respectively).

The ADC values of the MIBC and NMIBC tumors were (1.13 ± 0.31) × 10^−3^ mm^2^/s and (1.65 ± 0.42) × 10^−3^ mm^2^/s, respectively, the difference was statistically significant (*p* = 0.000). The ADC values of high- and low-grade tumors were (1.37 ± 0.39) × 10^−3^ mm^2^/s and (1.71 ± 0.42) × 10^−3^ mm^2^/s, respectively, and the difference was statistically significant (*p* = 0.003).

### 3.4. Diagnostic Performance of Multiple DKI Histogram Parameters

**Figure 3:**
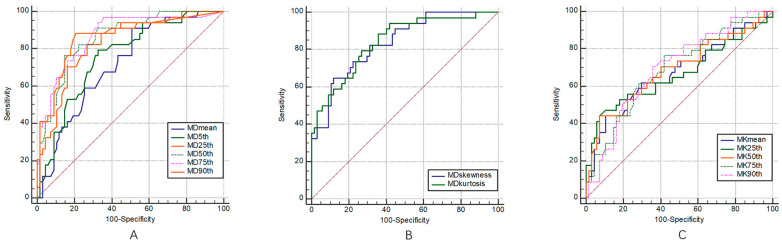
ROC curves of volumetric MD (**A**,**B**) and MK (**C**) histogram parameters for differentiating MIBC from NMIBC, respectively.

**Figure 4:**
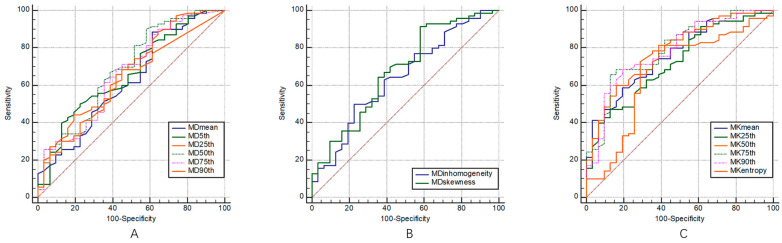
ROC curves of the volumetric MD (**A**,**B**) and MK (**C**) histogram parameters for differentiating low- from high-grade bladder cancer, respectively.

The ROC curves of the multiple DKI histogram parameters for the staging and grading of BC are shown in Figure 3 and Figure 4, and the corresponding diagnostic test characteristics are shown in Table 4 and Table 5.

In the identification of muscle invasiveness in bladder cancer, the AUC values of MDmean, MD5th, MD25th, MD50th, MD75th, MD90th, MDskewness, and MDkurtosis were 0.85, 0.76, 0.83, 0.86, 0.87, 0.87, 0.84, and 0.84, and the parameters of MD75th and MD90th yielded the highest AUCs of 0.87. The AUC values of MKmean, MK5th, MK25th, MK50th, MK75th, MK90th, MKskewness, and MKkurtosis were 0.67, 0.59, 0.67, 0.69, 0.69, 0.70, 0.61, and 0.53, respectively, and parameter of MK90th yielded the highest AUC of 0.70.

In the identification of the pathological grades of bladder cancer, the AUC values of MDmean, MD5th, MD25th, MD50th, MD75th, MD90th, MDinhomogeneity, MDskewness, and MDentropy were 0.68, 0.66, 0.67, 0.69, 0.66, 0.62, 0.64, 0.68, and 0.55, respectively, and the parameter of MD50th showed the highest diagnostic performance with an AUC of 0.69. The AUC values of MKmean, MK5th, MK25th, MK50th, MK75th, MK90th, MKinhomogeneity, MKskewness, and MKentropy were 0.77, 0.62, 0.72, 0.78, 0.79, 0.79, 0.53, 0.59, and 0.67, respectively, the parameters of MK75th and MK90th showed the highest diagnostic performance with an AUCs of 0.79.

### 3.5. Comparisons of the Diagnostic Performance of DKI Histogram Parameters and ADC Values, as Well as Biparametric VI-RADS

**Figure 5:**
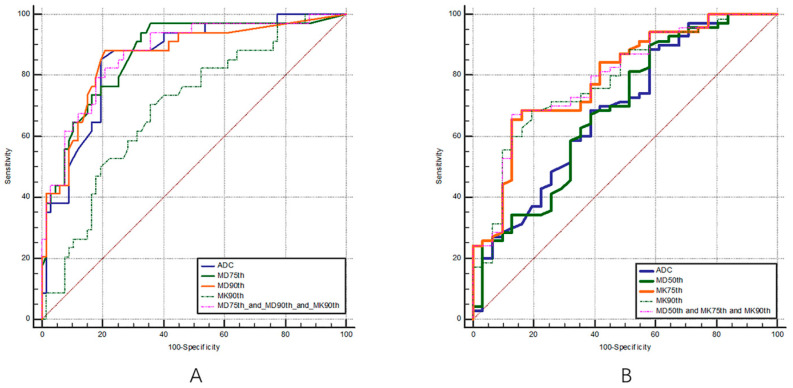
(**A**) ROC curves of the diagnostic performance of ADC, MD75th, MD90th, MK90th, and the combination of MD75th, MD90th, and MK90th for differentiating between MIBC and NMIBC; (**B**) ROC curves of the diagnostic performance of ADC, MD50th, MK75th, MK90th, and the combination of MD50th, MK75th, and MK90th for differentiating between high- and low-grade bladder cancer.

**Figure 6:**
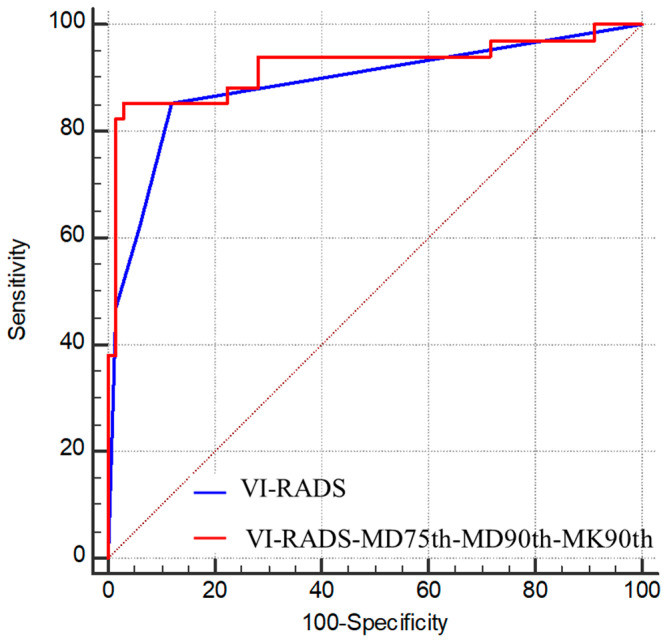
ROC curves of the diagnostic performance of VI-RADS score and the combination of MD75th, MD90th, and MK90th for differentiating between MIBC and NMIBC.

The ROC curves of the combination of DKI histogram parameters and ADC values of the stage and grade of BC are shown in Figure 5. The ROC curves of the combination of DKI histogram parameters and biparametric VI-RADS of stage of BC are shown in Figure 6.

In terms of staging, the AUC of MD75th (AUC = 0.87), MD90th (AUC = 0.87), and the combination of MD75th, MD90th, and MK90th (AUC = 0.87) were slightly higher than that of ADC (AUC = 0.86), but there was not significantly different (*p* = 0.375, 0.832, and 0.453, respectively). In terms of grading, the AUC of MK75th (AUC = 0.79), MK90th (AUC = 0.79), and the combination of MD50th, MK75th, and MK90th (AUC = 0.80) were significantly higher than that of ADC (AUC = 0.68) (*p =* 0.017, 0.040, and 0.018, respectively).

For inter-observer reliability of the biparametric VI-RADS scores between the two radiologists, the ICC was 0.868 with good consistency. The biparametric VI-RADS yielded a sensitivity of 85%, specificity of 88%, and AUC of 0.89 with a threshold score of 3 or higher for differentiating between NMIBC from MIBC. When combined with the MD and MK parameters (MD75th, MD90th, and MK90th), the AUC of biparametric VI-RADS improved to 0.92 for staging BC.

## 4. Discussion

Our study indicated that DKI derived histogram parameters had feasibility in differentiating MIBC from NMIBC, as well as low- from high-grade bladder cancer. Importantly, the diagnostic accuracy was improved with the combination of histogram parameters and biparametric VI-RADS in staging BC. Our study demonstrated the feasibility of using the TA of DKI diffusion model as a potential noninvasive tool to complement assessments based on histopathology and VI-RADS [23].

Previous studies have explored the value of DKI for the assessment of bladder cancer only using the conventional values of MK and MD. Our dates showed that the NMIBC group had higher MDmean, MD5th, MD25th, MD50th, MD75th, and MD90th than the MIBC group and lower MKmean, MK25th, MK50th, MK75th, and MK90th than the MIBC group, which were similar to the findings of Qing Li et al. [14] and Fang Wang et al. [24]. The results indicates that the degree of diffusion restriction is more in MIBC group, MIBC is heterogeneous with atypical cells and is characterized by vascular hyperplasia, endothelial proliferation, hemorrhage and necrosis [25], and they have more complex organizational structures and more heterogeneous in its distribution pattern. The higher MK value means the further deviation from Gaussian distribution and more factors that affect water diffusion. Namely, higher MK indicates higher cell density, more complex cell activity and more crowded intracellular environment. Our study showed that the high-grade group had lower MDmean, MD5th, MD25th, MD50th, MD75th and MD90th than the low-grade group and the high-grade group had higher MKmean, MK25th, MK50th, MK75th, MK90th, and MKentropy than the low-grade group, which were similar to the previous studies [26,27]. The results also showed that the degree of diffusion restriction is much higher in high-grade group, and its distribution pattern is also more heterogeneous and more complex. And the high-grade group had higher cell density, more complex cell activity and more crowded intracellular environment. However, Fang Wang et al. [13] found that only the MK values were significantly (*p* < 0.05) higher in patients of the MIBC group than in those of the NMIBC group. And only the MK values were significantly (*p* < 0.05) higher in patients with high-grade bladder tumors than in those with low-grade bladder tumors. Whereas MD values were not statistically different in differentiating bladder cancer stages and grades. This discrepancy may be attributed to the method of measurement (e.g., different ROI selection) and our application of texture analysis. Texture analysis is a tool for improving tumor heterogenicity, and this analysis more comprehensively estimates tumor biological [28,29]. Thus, our preliminary results showed that texture analysis with DKI functional maps based on the whole tumor, which was significantly superior to conventional DWI parameters in tumor grading and staging in bladder cancer. The results of this study are similar to the previous results of DKI in cervical cancer, which showed that the whole-tumor texture analysis of DKI can be used for differential diagnosis of cervical cancer subtypes and grading squamous cell cancer, and the diagnostic performance was better than that of conventional DWI [30].

Our results also showed that the MD values for the texture analysis had better diagnostic performance than the MK values in differentiating NMIBC from MIBC. In addition, the MK values for the texture analysis had better diagnostic performance than the MD values in differentiating low- from high-grade bladder cancer, which is similar to previous studies [26,27]. However, in a previous study [13], the AUC of the MD was 0.849 and the AUC of the MK was 0.905 in differentiating between MIBC and NMIBC. This discrepancy may be due to the differences in the research methods, scanning parameters, and sample sizes. And our study showed that the AUC of MD75th, MD90th, and the combination of MD75th, MD90th, and MK90th were slightly higher than ADC in differentiating MIBC from NMIBC, and the AUC of MK75th, MK90th, and the combination of MD50th, MK75th, and MK90th were significantly higher than ADC in differentiating low- from high-grade bladder cancer. Compared with conventional DWI, DKI can provide more quantitative parameters to reflect the changes of tumor microstructure and better reflect the complexity of tumor microstructure [31,32]. Furthermore, texture analysis is a useful additional tool for tumor imaging, providing a range of quantitative parameters to better reflect tumor heterogeneity [28,29,33]. These may be the reasons why the whole-tumor texture analysis applied to DKI can produce a more robust value than conventional ADC value in distinguishing NMIBC from MIBC and low- from high-grade bladder cancer.

In 2018, Panebianco et al. [7] reported that VI-RADS score have been widely used to assess the stage of bladder cancer. However, the VI-RADS score is a qualitative assessment with a certain human factor and is relatively not so stable. Our study has showed that the combination of the TA of DKI parameters and VI-RADS can improve the performance of VI-RADS by simultaneously quantifying the characteristics of tissue structure, including tumor microstructure and heterogeneity.

This study had several limitations. First, the number of cases is moderate, the number of different stages and grades of bladder cancer is uneven, and the number of cases of NMIBC and high-grade bladder cancer is higher, which may lead to statistical bias. In addition, further study of a larger population was needed. Second, as a single-center retrospective study, there may be a flaw in selection bias. Third, for patients with multiple bladder cancers, we only selected the largest lesion for analysis, which may also lead to selection bias. Lastly, our study only performed a biparametric VI-RADS score because some patients lacked a DCE sequence; However, biparametric VI-RADS and full VI-RADS scores have been documented to be of considerable value [34].

## 5. Conclusions

In conclusion, our study demonstrated that both volumetric DKI histogram analysis and conventional DWI could be used to assess muscle invasion and histologic grade of bladder cancer. Furthermore, the texture analysis of DKI model parameters outperformed the conventional ADC. The diagnostic performance could be improved when combining the histogram parameters of DKI (MD75th, MD90th, and MK90th) and biparametric VI-RADS.

## Figures and Tables

**Table 1:** Characteristics of patients with bladder cancer.

Characteristic		No. (%)
age (year)	63.5 ± 9.8 (36–84)	
gender	male	87
	female	14
diameter	≤3 cm	75
	>3 cm	26
stage	≤T1	67
	≥T2	34
grade	high grade	70
	low grade	31
surgical method	TURBT	69
	radical or partial bladder resection	32

**Table 2:** Comparisons of the MD and MK histogram parameters values between MIBC and NMIBC.

	MD			MK		
	NMIBC	MIBC	*p*	NMIBC	MIBC	*p*
mean	1.92 ± 0.38	1.42 ± 0.34	0.00	0.69 ± 0.16	0.81 ± 0.20	0.00
5th	1.19 ± 0.39	0.91 ± 0.25	0.00	0.38 ± 0.50	0.38 ± 0.27	0.99
25th	1.53 ± 0.38	1.11 ± 0.28	0.00	0.60 ± 0.15	0.69 ± 0.22	0.04
50th	1.86 ± 0.42	1.31 ± 0.33	0.00	0.72 ± 0.15	0.84 ± 0.20	0.00
75th	2.75 ± 0.33	1.63 ± 0.46	0.00	0.81 ± 0.18	0.96 ± 0.22	0.00
90th	2.75 ± 0.33	2.06 ± 0.56	0.00	0.92 ± 0.33	1.06 ± 0.25	0.04
inhomogeneity	0.29 ± 0.08	0.32 ± 0.09	0.12	0.38 ± 0.76	0.29± 0.16	0.52
skewness	0.43 ± 0.63	1.31 ± 0.69	0.00	−1.05 ± 0.91	−0.78 ± 0.72	0.13
kurtosis	−0.37 ± 1.04	2.45 ± 3.41	0.00	2.25 ± 2.71	1.76 ± 2.21	0.33
entropy	3.88 ± 0.40	3.89 ± 0.26	0.86	3.78 ± 0.25	3.84 ± 0.25	0.25

The units of the mean, 5th, 25th, 50th, 75th, 90th of the MD group are 10^−3^.

**Table 3:** Comparisons of the MD and MK histogram parameters values between the high- and low-grade bladder cancer.

	MD			MK		
	High Grade	Low Grade	*p*	High Grade	Low Grade	*p*
mean	1.66 ± 0.41	1.97 ± 0.44	0.00	0.78 ± 0.17	0.61 ± 0.15	0.00
5th	1.03 ± 0.32	1.27 ± 0.42	0.00	0.43 ± 0.49	0.29 ± 0.24	0.14
25th	1.30 ± 0.34	1.59 ± 0.47	0.00	0.67 ± 0.17	0.53 ± 0.16	0.00
50th	1.56 ± 0.41	1.92 ± 0.51	0.00	0.81 ± 0.17	0.64 ± 0.13	0.00
75th	1.99 ± 0.54	2.34 ± 0.54	0.00	0.92 ± 0.19	0.73 ± 0.15	0.00
90th	2.45 ± 0.56	2.68 ± 0.42	0.04	1.04 ± 0.33	0.80 ± 0.18	0.00
inhomogeneity	0.31 ± 0.09	0.27 ± 0.08	0.01	0.37 ± 0.75	0.31 ± 0.15	0.64
skewness	0.88 ± 0.74	0.37 ± 0.74	0.00	−0.84 ± 0.77	−1.22 ± 1.00	0.06
kurtosis	0.91 ± 2.82	−1.47 ± 1.44	0.05	1.81 ± 2.02	2.73 ± 3.42	0.17
entropy	3.93 ± 0.26	3.77 ± 0.50	0.10	3.83 ± 0.25	3.72 ± 0.23	0.04

The units of the mean, 5th, 25th, 50th, 75th, 90th of the MD group are 10^−3^.

**Table 4:** Diagnostic performances of the MD and MK histogram parameters values for differentiating between MIBC and NMIBC.

	MD					MK				
	AUC	Cut-Off Value	95% CI	Sensitivity	Specificity	AUC	Cut-Off Value	95% CI	Sensitivity	Specificity
mean	0.85	1.63	0.76 to 0.91	73.5	85.1	0.67	0.87	0.57 to 0.76	44.1	89.6
5th	0.76	1.05	0.66 to 0.84	79.4	67.2	0.59	0.52	0.49 to 0.69	44.1	77.6
25th	0.83	1.33	0.74 to 0.89	82.4	73.1	0.67	0.76	0.57 to 0.76	47.1	91.0
50th	0.86	1.51	0.78 to 0.92	76.5	85.1	0.69	0.90	0.59 to 0.78	44.1	92.5
75th	0.87	2.14	0.79 to 0.93	94.1	67.2	0.69	0.84	0.59 to 0.78	76.5	58.2
90th	0.87	2.53	0.79 to 0.93	88.2	80.1	0.70	0.93	0.60 to 0.78	70.1	64.2
skewness	0.84	1.11	0.75 to 0.90	64.7	88.1	0.61	−1.26	0.51 to 0.70	82.4	44.8
kurtosis	0.84	−0.08	0.76 to 0.91	79.4	73.1	0.53	2.30	0.43 to 0.63	79.4	40.3

**Table 5:** Diagnostic performances of the MD and MK histogram parameters values for differentiating between low- and high-grade bladder cancer.

	MD					MK				
	AUC	Cut-Off Value	95% CI	Sensitivity	Specificity	AUC	Cut-Off Value	95% CI	Sensitivity	Specificity
mean	0.68	2.08	0.58 to 0.77	88.6	41.9	0.77	0.73	0.67 to 0.84	58.6	80.1
5th	0.66	1.02	0.56 to 0.75	51.4	77.4	0.62	0.54	0.52 to 0.71	31.4	90.3
25th	0.67	1.67	0.57 to 0.76	87.1	38.7	0.72	0.69	0.62 to 0.81	47.1	87.1
50th	0.69	2.07	0.59 to 0.78	91.4	41.9	0.78	0.75	0.69 to 0.86	60.0	83.9
75th	0.66	2.26	0.56 to 0.75	68.6	58.1	0.79	0.85	0.70 to 0.87	65.7	87.1
90th	0.62	2.76	0.52 to 0.72	65.7	58.1	0.79	0.79	0.69 to 0.86	65.7	83.9
inhomogeneity	0.64	0.31	0.54 to 0.73	50.0	77.4	0.53	0.43	0.43 to 0.63	91.4	29.0
skewness	0.68	0.16	0.58 to 0.77	91.4	41.9	0.59	−1.80	0.49 to 0.69	95.7	35.5
entropy	0.55	3.60	0.45 to 0.65	88.6	32.3	0.67	3.78	0.57 to 0.76	72.9	71.0

## Data Availability

The data is not publicly available, but authors can be contacted privately if requested.

## Supplementary Materials

The following supporting information can be downloaded at: https://www.mdpi.com/article/10.3390/bioengineering10070745/s1, Figure S1: Five-point VI-RADS scoring system based on T2WI and DWI; Table S1: Measurement consistency of two observers of DKI histogram parameter.
