# Supplementary material for: Evaluation of Whole-Tumor Texture Analysis Based on MRI Diffusion Kurtosis and Biparametric VI-RADS Model for Staging and Grading Bladder Cancer
Source: Bioengineering (Basel). 2023 Jun 21;10(7):745. doi: 10.3390/bioengineering10070745 (PMC10376391; doi:10.3390/bioengineering10070745)
Supplement: Supplementary file 1 [file bioengineering-10-00745-s001.zip › bioengineering-2431301-supplementary.pdf]

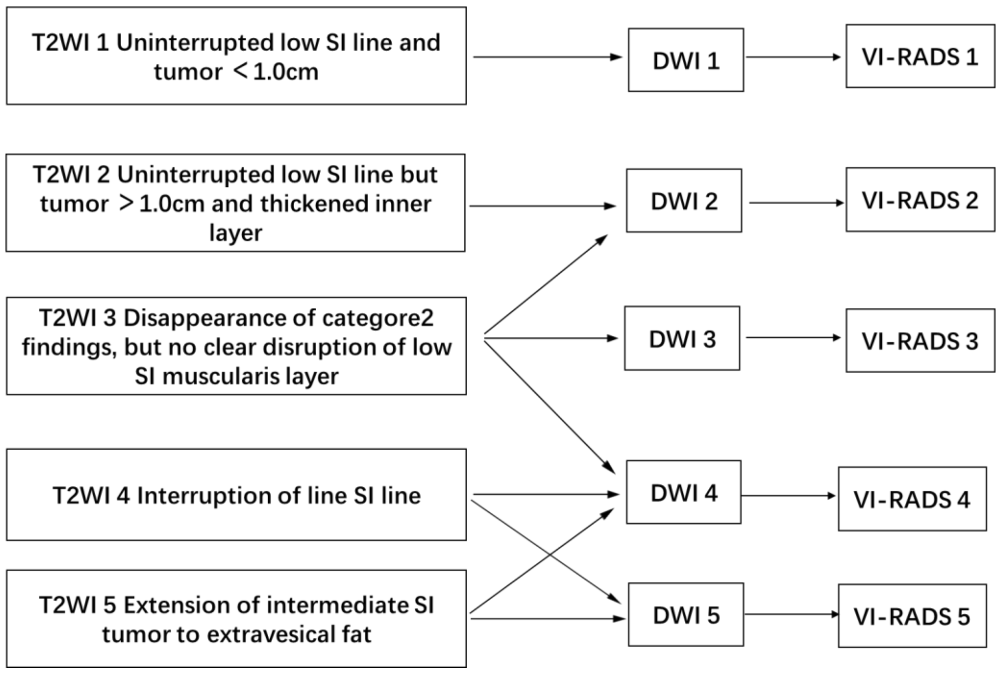

Figure S1: Five-point VI-RADS scoring system based on T2WI and DWI.

Table S1: Measurement consistency of two observers of DKI histogram parameter.

	MD		MK	
	ICC	95%CI	ICC	95%CI
mean	0.980	0.957 to 0.990	0.992	0.992 to 0.998
5th	0.997	0.993 to 0.999	0.984	0.967 to 0.993
25th	0.997	0.993 to 0.998	0.984	0.966 to 0.992
50th	0.993	0.985 to 0.997	0.997	0.993 to 0.998
75th	0.970	0.938 to 0.986	0.998	0.996 to 0.999
90th	0.910	0.811 to 0.957	0.993	0.986 to 0.997
inhomogeneity	0.961	0.919 to 0.981	0.992	0.984 to 0.996
skewness	0.928	0.849 to 0.966	0.958	0.912 to 0.980
kurtosis	0.900	0.790 to 0.953	0.922	0.834 to 0.963
entropy	0.956	0.907 to 0.979	0.915	0.818 to 0.959
